# Supplementary material for: Development of a computer-aided design software for the quantitative evaluation of aesthetic damage
Source: PLoS One. 2019 Dec 18;14(12):e0226322. doi: 10.1371/journal.pone.0226322 (PMC6919621; doi:10.1371/journal.pone.0226322)
Supplement: S1 Code site — 1. (ZIP) [file pone.0226322.s001.zip › [EESC_jr][Projeto]Relatorio_07_21/reports/templates_zip/gentelella-master.zip!/gentelella-master/production/e_commerce.html]

Gentellela Alela! | 


Gentellela Alela!

Welcome,

## John Doe

  


### General

- Home 
  - Dashboard
  - Dashboard2
  - Dashboard3
- Forms 
  - General Form
  - Advanced Components
  - Form Validation
  - Form Wizard
  - Form Upload
  - Form Buttons
- UI Elements 
  - General Elements
  - Media Gallery
  - Typography
  - Icons
  - Glyphicons
  - Widgets
  - Invoice
  - Inbox
  - Calendar
- Tables 
  - Tables
  - Table Dynamic
- Data Presentation 
  - Chart JS
  - Chart JS2
  - Moris JS
  - ECharts
  - Other Charts
- Layouts 
  - Fixed Sidebar
  - Fixed Footer

### Live On

- Additional Pages 
  - E-commerce
  - Projects
  - Project Detail
  - Contacts
  - Profile
- Extras 
  - 403 Error
  - 404 Error
  - 500 Error
  - Plain Page
  - Login Page
  - Pricing Tables
- Multilevel Menu 
  - Level One- Level One
      - Level Two
      - Level Two
      - Level Two
    - Level One
- Landing Page Coming Soon

- John Doe
  - Profile
  - 50%
    Settings
  - Help
  - Log Out
- 6
  - John Smith
    3 mins ago

    Film festivals used to be do-or-die moments for movie makers. They were where...
  - John Smith
    3 mins ago

    Film festivals used to be do-or-die moments for movie makers. They were where...
  - John Smith
    3 mins ago

    Film festivals used to be do-or-die moments for movie makers. They were where...
  - John Smith
    3 mins ago

    Film festivals used to be do-or-die moments for movie makers. They were where...
  - **See All Alerts**


### E-commerce :: Product Page

Go!

## E-commerce page design

- - Settings 1
  - Settings 2

### LOWA Men’s Renegade GTX Mid Hiking Boots Review

Raw denim you probably haven't heard of them jean shorts Austin. Nesciunt tofu stumptown aliqua butcher retro keffiyeh dreamcatcher synth. Cosby sweater eu banh mi, qui irure terr.

  

## Available Colors

- Green
- Blue
- Red
- Orange

  

## Size Please select one

- Small
- Medium
- Large
- Xtra-Large

  

# Ksh80.00

Ex Tax: Ksh80.00

Add to Cart
Add to Wishlist

- Home
- Profile
- Profile

Raw denim you probably haven't heard of them jean shorts Austin. Nesciunt tofu stumptown aliqua, retro synth master cleanse. Mustache cliche tempor, williamsburg carles vegan helvetica. Reprehenderit butcher retro keffiyeh dreamcatcher
synth. Cosby sweater eu banh mi, qui irure terr.

Food truck fixie locavore, accusamus mcsweeney's marfa nulla single-origin coffee squid. Exercitation +1 labore velit, blog sartorial PBR leggings next level wes anderson artisan four loko farm-to-table craft beer twee. Qui photo
booth letterpress, commodo enim craft beer mlkshk aliquip

xxFood truck fixie locavore, accusamus mcsweeney's marfa nulla single-origin coffee squid. Exercitation +1 labore velit, blog sartorial PBR leggings next level wes anderson artisan four loko farm-to-table craft beer twee. Qui
photo booth letterpress, commodo enim craft beer mlkshk


Gentelella - Bootstrap Admin Template by Colorlib
